# Association of telomerase activity with radio- and chemosensitivity of neuroblastomas

**DOI:** 10.1186/1748-717X-5-66

**Published:** 2010-07-19

**Authors:** Simone Wesbuer, Claudia Lanvers-Kaminsky, Ines Duran-Seuberth, Tobias Bölling, Karl-Ludwig Schäfer, Yvonne Braun, Normann Willich, Burkhard Greve

**Affiliations:** 1 Department of Radiotherapy -Radiooncology-, University Hospital Münster, Albert-Schweitzer-Straße 33, D-48149 Münster; 2 Department of Paediatric Haematology and Oncology, University Hospital, Münster, Germany; 3 Institute of Pathology, Heinrich-Heine University Düsseldorf, Germany

## Abstract

**Background:**

Telomerase activity compensates shortening of telomeres during cell division and enables cancer cells to escape senescent processes. It is also supposed, that telomerase is associated with radio- and chemoresistance. In the here described study we systematically investigated the influence of telomerase activity (TA) and telomere length on the outcome of radio- and chemotherapy in neuroblastoma.

**Methods:**

We studied the effects on dominant negative (DN) mutant, wild type (WT) of the telomerase catalytic unit (hTERT) using neuroblastoma cell lines. The cells were irradiated with ^60^Co and treated with doxorubicin, etoposide, cisplatin and ifosfamide, respectively. Viability was determined by MTS/MTT-test and the GI_50 _was calculated. Telomere length was measured by southernblot analysis and TA by Trap-Assay.

**Results:**

Compared to the hTERT expressing cells the dominant negative cells showed increased radiosensitivity with decreased telomere length. Independent of telomere length, telomerase negative cells are significantly more sensitive to irradiation. The effect of TA knock-down or overexpression on chemosensitivity were dependent on TA, the anticancer drug, and the chemosensitivity of the maternal cell line.

**Conclusions:**

Our results supported the concept of telomerase inhibition as an antiproliferative treatment approach in neuroblastomas. Telomerase inhibition increases the outcome of radiotherapy while in combination with chemotherapy the outcome depends on drug- and cell line and can be additive/synergistic or antagonistic. High telomerase activity is one distinct cancer stem cell feature and the here described cellular constructs in combination with stem cell markers like CD133, Aldehyddehydrogenase-1 (ALDH-1) or Side population (SP) may help to investigate the impact of telomerase activity on cancer stem cell survival under therapy.

## Background

Telomeres are special structures at the end of chromosomes, which comprise repetitive DNA-sequences ((TTAGGG)n) combined with distinct proteins. They protect chromosomes from end-to-end fusions and from loosing coding sequences during mitosis. They are 15-20 kB in length and are shortened in the range of 20 to 200 basepairs with each cell cycle and by this preventing loss of coding DNA-sequences and end to end fusion of chromosomes during cell cycle. If telomere length reaches a critical length, cells become senescent. Thus telomeres serve as a mitotic clock and determine senescence processes.

The telomeric sequence is a structural feature of all cells but some have the potential to recover telomere length by the activity of the enzyme telomerase, a ribonucleoprotein-complex which elongates telomeric sequences by its internal RNA-template and which is expressed preferentially in germ cells, stem cells or activated lymphocytes. However, it is well known, that more than 90% of all human malignant tumor entities reactivate telomerase activity [1] and especially cancer stem cells are reported to have the potential to recover high telomerase activity [2,3]. By reactivation, tumor cells achieve the ability for unlimited proliferation during carcinogenesis [4-6]. In this way, telomerase is expected to be a promising target in malignant tumor treatment and a prognostic marker in tumor progression and therapeutic response [7].

Current literature indicates a relationship between cellular radiosensitivity and telomere length [8-10]. Goytisolo et al. reported a clear synergistic effect of telomerase inhibition, telomere shortening and radiation response of normal tissue [11]. These findings were confirmed by Wong et al. investigating telomere length and radiosensitivity in knock-out mice [12]. Irradiation and chemotherapy also seem to modulate telomerase activity and human telomerase reverse transcriptase (hTERT) gene expression in vitro and in xenograft-tumors in vivo [13-16]. Inhibition of telomerase has a significant influence on cell death processes and was reported to increase apoptosis probably by loss of chromosomal T-loop protection [17]. Accordingly, it would be of high interest to know whether the modulation of telomerase activity has an impact on radio- and chemotherapy or not especially in those tumors with high telomerase expression and high radioresistance which both are also distinctive freatures of cancer stem cells [2,18].

Therefore, we transformed different cell lines of a tumor which was described to be radioresistant (Neuroblastoma) [19] with vectors which either lead to a stable overexpression or to a complete downregulation of telomerase activity. These cells were used as models to investigate the influence of telomerase activity as well as telomere length on the outcome of chemo- and/or radiotherapy.

## Methods

### Cell transformation

The neuroblastoma cell lines CHLA-90 and SK-N-SH were transfected. CHLA-90 was kindly provided from C.P. Reynolds, Division of Hematology-Oncology, USC-CHLA Institute for Pediatric Clinical Research, Children's Hospital Los Angeles, Los Angeles, USA). SK-N-SH was purchased from the American Tissue Culture Collection, Promochem). All cell lines were of polyclonal origin.

### Cell culture

The cells were grown in RPMI1640 cell culture medium supplemented with 10% fetal calf serum, 2 mmol/L L-glutamine, penicillin and streptomycin. Cells were passaged twice a week and used for drug treatment and irradiation after 20 to 22 population doublings. The dominant negative SK-N-SH cells survive only a limited number of doublings. For viability tests cells were transferred onto 96 well plates with a density of 5,000 cells per well. After 72 h cells were either irradiated with 1, 2, 5, 10, 20 Gy X-ray (Telekobalt Phillips, Hamburg, Germany) or exposed to 2.5 × 10^-6 ^- 2.5 × 10^-10 ^mol/L doxorubicin (Adriblastin™, Pharmacia, Karlsruhe, Germany), 1 × 10^-4 ^- 1 × 10^-8 ^mol/L etoposide (Eto-GRY™, Gry-Pharma, Kirchzarten, Germany), 1 × 10^-4 ^- 1 × 10^-8 ^mol/L cisplatin (Platinex™, Bristol-Myer Squibb, München, Germany), 1 × 10^-4 ^- 1 × 10^-8 ^mol/L 4-Hydroxy-peroxy-ifosfamide (ASTA, Frankfurt, Germany). Cell viability was analysed after 24 h, 48 h, 72 h, and 96 h using the MTS or MTT assay. Experiments were carried out in quadruplate and each experiment was repeated independently three times. From each MTS/MTT experiment aliquots of cells were frozen in liquid nitrogen for telomere length and telomerase activity measurements.

### MTS-Test

After treatment cell viability was determined after 24 h, 48 h, 72 h, and 96 h by the MTS or the MTT assay as described previously [20].

The MTT and MTS assay base on the same principle. Both rely on the formation of a purple formazan dye by mitochondrial aldehyd dehydrogenases of viable cells. The formazan dye formed from MTS is water soluble and can be determined spectrophotometrically 3 h after MTS addition at a wavelength of 490 nm using a microplate reader (BioRad Laboratories, München, Germany). Since the colour of test drugs like doxorubicin might interfere with the absorption of the MTS formazan, the in vitro tests of anticancer drugs was performed with the MTT test, while the cytotoxicity of irradiation was determined by the MTS assay. The formazan crystals formed from the MTT reagent are not water soluble. Therefore, 3 h after addition of the MTT reagent the supernatant was removed and the blue formazan crystals were dissolved in a solution consisting of 20% (g/v) sodium dodecylsulphate (SDS) and a mixture of demineralised water and dimethylformamide (1:1) and its color was quantified spectrophotometrically at a wavelength of 560 nm with an Ascent Multiscan^® ^microplate reader (Thermo Fisher Scientific, Langenselbold, Germany).

The optical densities were used to determine the drug concentration that reduces the activity of mitochondrial aldehyde dehydrogenases by 50% compared to that observed in control cells incubated for 72 h without test drug (GI_50_).

### Southernblot analysis

After cell lysis genomic DNA was extracted by conventional phenol-chloroform method [21]. Telomere length was determined by telomere restriction fragment assay (TRF) using the TeloTAGGG Telomere Length Assay Kit (Roche, Grenzach-Wyhlen, Germany). In detail, 1 μg purified DNA was digested by 20 units of RsaI and HinfI for 2 h at 37°C. Gel eletrophoresis was carried out on a 1% agarose gel with 50 V for 16 h at 4°C. After HCl treatment, denaturation and neutralization, DNA-fragments were transferred to nylon membrane by capillarity for 16 h at room temperature. The transferred DNA was fixed by heating the membrane to 120°C for 20 minutes. The hybridization was carried out with DIG-conjugated telomeric probe for 3 h at 42°C. Finally, the membrane was washed twotimes and labelled with anti-DIG-AP antibody. The telomeres were visualized by chemiluminiscence. Telomere length was determined by using the program Telorun.

### Trap-Assay

**Figure 1:**
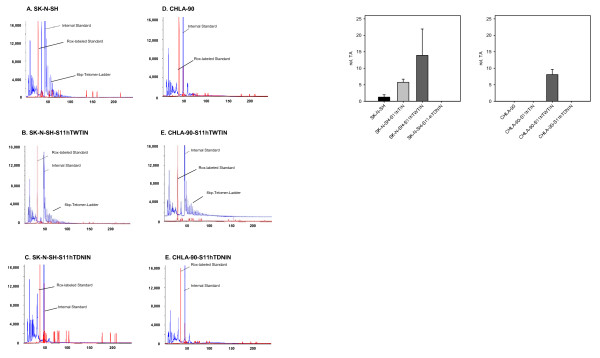
**Determination of telomerase activity.** A. Telomerase activity of transfected and not-transfected CHLA-90 and SK-N-SH cells as determined by the TRAP assay (SK-N-SH and CHLA-90: non-transfected cell lines; SK-N-SH-S11hTWTI and CHLA-90-S11hTWTI: overexpressing cell lines; SK-NSH-S11hTDNI and CHLA-90-S11hTDNI: knockdown cell lines). B. Mean relative Telomerase activity of transfected and not-transfected CHLA-90 and SK-N-SH cells as determined by the TRAP assay from three different passages.

Telomerase activity was determined by a modified TRAP (Telomeric Repeat Amplification Protocol) assay, using the TRAPeze kit (Chemicon International, Germany). In the first step of the TRAP assay, telomerase of cell lysates added hexamer repeats of telomeric sequence (TTAGGG) onto the 3'-end of an included oligonucleotide. Subsequently the synthesized telomeric repeats were amplified by *Taq*-polymerase in a regular polymerase chain reaction in the presence of a fluorescent 6-carboxyfluorescein (6-FAM)-labelled TS primer. The resulting PCR products of 50, 56, 62, 68, etc. base pairs generated a characteristic ladder with six pair increments when separated by capillary electrophoresis (ABI 3730, Applied Biosystems, Germany) (Fig 1).

### Transfection

For transfection the retroviral vector S11IN was used, which was kindly provided by Dr. Helmut Haneberd (Dept. of Pediatric Oncology, University of Duesseldorf, Germany). The S11IN vectors containing wild type and mutant hTERT were constructed by subcloning the respective hTERT (T) cDNA sequence of the wild-type (WT) and the mutant hTERT (DN, dominant negative) from the pBABE-puro DN plasmid and the pBABE-puro WT plasmid (kind gifts of Dr. Robert A. Weinberg, Whitehead Institute, Cambridge, USA) using standard protocols. Selection of S11hTDNIN and S11hTWTIN transfected cells was carried out with geneticin (G418 sulfate) (Invitrogen, Karlsruhe, Germany). Confirmation of pS11 contruction insertion was proofed by PCR analysis and DNA sequencing. In addition to the S11hTDNIN and S11hTWTIN cells were also transfected with S11IN vector in order to characterise the effect of vector transfection alone on proliferation, viability, chemo- and radiosensitivity.

### Statistics

GI_50 _is the drug concentration that reduces the activity of mitochondrial aldehyde dehydrogenases by 50% compared to that observed in control cells incubated for 72 h without test drug. For the calculation of GI_50_s the following formula was used: (50% - [% viable cells (< 50%)])/([% viable cells (> 50%)] - [% viable cells (< 50%)]) * (drug concentration > 50% viable cells - drug concentration < 50% viable cells) + (drug concentration < 50% viable cells). Significance was determined by using the One-Way ANOVA -Holm-Sidiak method, p < 0.05 (Sigma Plot 11.0, systat.com) All experiments were done in triplicates.

## Results

### Transfected cell lines

**Figure 2:**
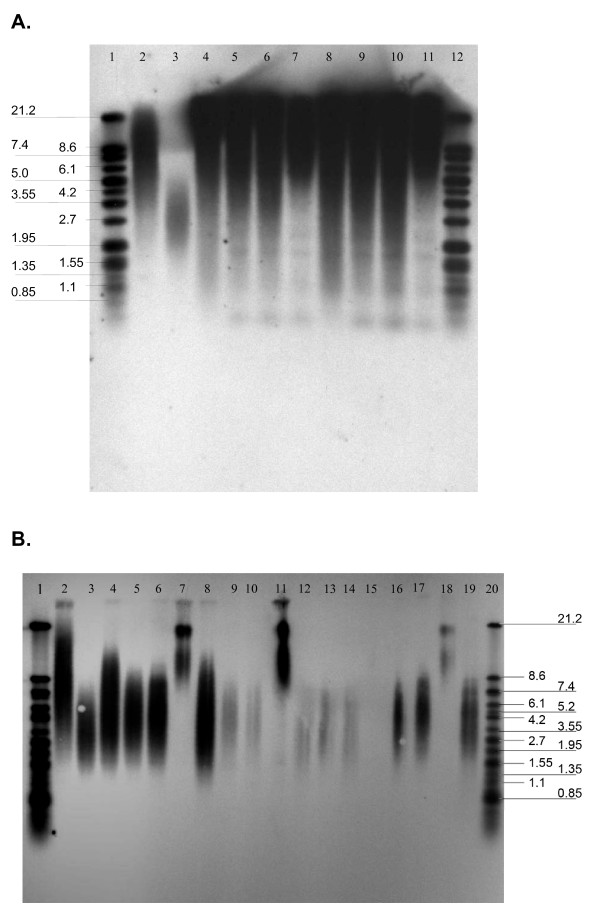
**Determination of telomere length.** A. Telomere length southern of transfected and not-transfected CHLA-90 cells. (1. DIG weight marker; 2. DNA high: 5.5 kb; 3. DNA low: 3.2 kb; 4. CHLA-90 4.7 kb; 5. CHLA-90-IN (passage 41) 4.8 kb; 6. CHLA-90-hTDNIN (passage: 39) 4.7 kb; 7. CHLA-90-hTWTIN (passage 42) 5.5 kb; 8. CHLA-90: 3.9 kb; 9. CHLA-90-IN (passage 40) 4.0 kb; 10. CHLA-90-hTDNIN (passage 42) 3.6 kb; 11. CHLA-90-hTWTIN (passage 45) 4.7 kb; 12. DIG weigth marker. B. Telomere length southern of transfected and not-transfected SK-N-SH cells. (1. DIG weight marker; 2. DNA high: 6.7 kb; 3. DNA low: 3.6 kb; 4. SK-N-SH: 4.7 kb, 5. SK-N-SH-IN (passage 20): 4.3 kb; 6. SK-N-SH-hTDNIN (passage 21): 4.3 kb; 7. SK-N-SH-hTWTIN (passage 21) 15 kb; 8. SK-N-SH: 3.8 kb; 9. SK-N-SHIN (passage 22): 4.9 kb; 10. SK-N-SH-hTDNIN (passage 23) 6.2 kb; 11. SK-N-SH-hTWTIN (passage 23): 14.2 kb; 12. SK-N-SH: 4.3 kb; 13. SK-N-SH-IN (passage 28): 4.7 kb; 14. SK-N-SH-hTDNIN (passage 26): 4.7 kb; 15. SK-N-SH-hTWTIN (passage 29) not evaluable; 16. SK-N-SH-IN (passage 20) 4.3 kb; 17. SK-N-SH-hTDNIN (passage 21) 4.6 kb; 18. SK-N-SH-hTWTIN (passage 21) 16.7 kb; 19. SK-N-SH-hTDNIN (passage 27) 3.2 kb; 20. DIG weight marker).

To study the effect of TA on radio- and chemosensitivity of neuroblastomas two neuroblastoma cell lines, CHLA-90 and SK-N-SH were stably transfected with wild-type hTERT and a dominant negative mutant of hTERT. Telomerase was present in the neuroblastoma cell line SK-N-SH, while no TA was detected in CHLA-90 cells (Fig. 1). These cells overcome telomere erosion during cell division by an alternative lengthening of telomeres (ALT), which is characterized by a broad range of telomere length within these cells (Fig. 2).

The dominant negative hTERT mutant completely blocked TA activity in the TA positive cell line SK-N-SH (Fig. 1). Transfection with wild-type hTERT increased the relative TA in SK-N-SH more than 10-fold. Moreover, with increasing population doublings the knock-down of hTERT resulted in gradual telomere erosion of S11hTDNIN transfected SK-N-SH, while overexpression of wild-type hTERT significantly increased the telomere length of transfected cells (Fig. 2). SK-N-SH cells transfected with the dominant negative hTERT mutant initially showed the same growth characteristics compared to not transfected cell lines. However, after more than 28 passages along with telomere shortening cell growth slowed down. The cells finally detached from the tissue culture flask and died. Transfection of SK-N-SH with S11hTWTIN and S11IN, however, did not influence cell proliferation.

Though transfection of TA-negative CHLA-90 cells with wild-type hTERT rendered these cells TA positive (Fig. 1) and resulted in an increase of telomere length (Fig. 2), it had no effect on the proliferation of these cell lines. In addition, transfection of CHLA-90 with the dominant-negativ hTERT mutant nor with the S11IN vector affected cell proliferation.

### Radiotherapy

**Figure 3:**
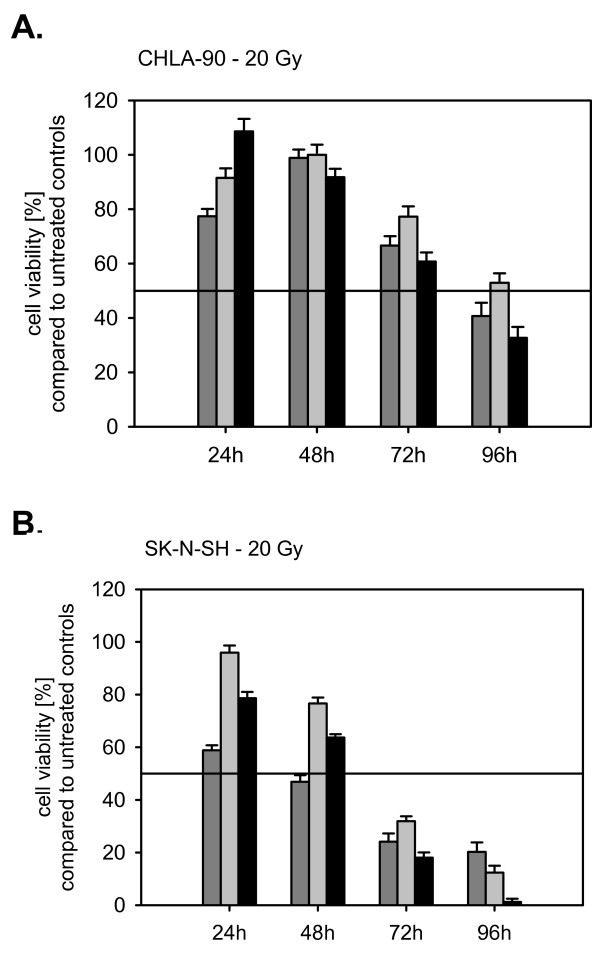
**Cytotoxicity of irradiation on S11hTDNIN (Black line), S11hTWTIN (Grey line), and S11hTIN (Dark grey line) transfected CHLA-90 (A.) and SK-N-SH (B.) 24 h, 48 h, 72 h, and 96 h post irradiation**.

Radiation reduced cell viability of the neuroblastoma cell lines with increasing radiation dosage. The cytotoxicity observed increased with increasing post irradiation interval. CHLA-90 cells were more radioresistant than SK-N-SH cells. For the neuroblastoma cell lines an inverse relationship between TA expression and radiosensitivity was observed. Knocking down TA in the TA-expressing SK-N-SH cell line increased the radiosensitivity of these cells compared to S11hTWTIN transfected cells (Fig. 3). On the other hand expression of TA in TA-negative CHLA-90 cells decreased the radiosensitivity (Fig. 3). Both, the radioprotective effect of ektope TA expression as well as the radiosensitizing effect became more prominent after longer post irradiation intervals. The differences were consistently significant for all time points.

### Chemotherapy

All anticancer drugs reduced cell viability of transfected and not-transfected cell lines in a time and dose dependent manner. The effects of TA knock-down or overexpression on chemosensitivity and -resistance were dependent on TA, the anticancer drug, and the chemosensitivity of the maternal cell line.

**Figure 4:**
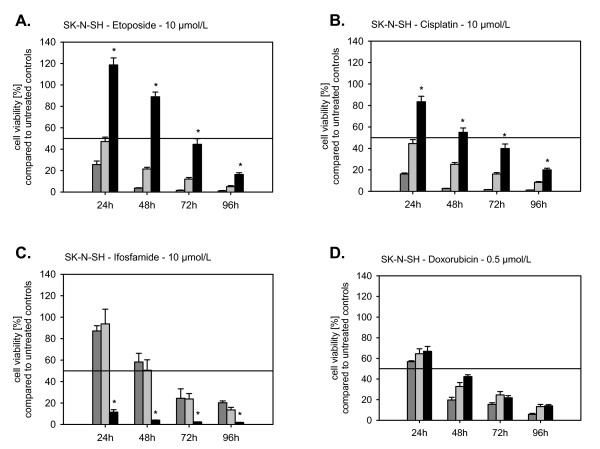
**Cytotoxicity of etoposide (A.), cisplatin (B.), ifosfamide (C.), and doxorubicin (D.) on S11hTDNIN (Black line), S11hTWTIN (Grey line), and S11hTIN (Dark grey line) transfected SK-N-SH cells after 24 h, 48, 72 h, and 96 h**.

Transfection of wild-type and dominant negative hTERT modulated the chemosensitivity of SK-N-SH cells. The dominant negative transfected hTERT cell lines became significantly more resistant to cisplatin, etoposide, and doxorubicin. However, transfection with dominant negative hTERT rendered the SK-N-SH more sensitive against ifosfamide (Fig. 4). Modulation of drug sensitivity/resistance was most prominent after drug exposure for 24 h. The differences between transfected and not-transfected cell lines declined with increasing duration of drug exposure (Fig.4).

Transfection of CHLA-90 only slightly modulated the sensitivity against cisplatin, ifosfamide, doxorubicin, and etoposide. Since there was less than two fold difference between different transfected clones, these effects were not considered significant

## Discussion

The introduction of chemotherapy and radiotherapy combined with tumor resection significantly improved treatment outcome of children suffering from neuroblastomas [22]. However, despite of all further efforts within recent years the prognosis of patients with advanced and/or disseminated disease is still poor, demonstrating the need of new therapeutic approaches for these patients [23-26].

During tumorigenesis the enzyme telomerase is reactivated in the fast majority of these tumors promoting tumor growth and aggressiveness [27,28]. Since telomerase is almost exclusively expressed at high levels in most tumors it is a promising selective target for the treatment of cancer. Hahn et al. at first demonstrated that telomerase inhibition of telomerase expressing human tumor cells effectively inhibited tumor growth [29].

Establishing stable transfected cell lines we were able to verify this concept for neuroblastomas, too. However, inhibition of tumor growth as a consequence of telomerase inhibition only occurs after an appropriate number of cell divisions, when the telomeres reach a critical length and tumor cells consequently enter a state of senescence. Thus, telomerase inhibition alone is not a promising approach, but it might add benefits, when combined with chemotherapy or irradiation. We decided to use the stable transfected cell lines to study the effects of telomerase inhibition on chemo- and radiosensitivity of neuroblastomas, since small molecules, which inhibit TA i.e. by stabilizing the G-quadruplex structure of telomeres, despite of high selectivity are likely to exert off target effects, too. As standard anticancer drugs doxorubicin, etoposide, cisplatin, and ifosfamide were chosen, which are well established in the treatment of neuroblastomas.

For irradiation there was an inverse relationship between TA expression and radiosensitivity. Ektope expression of TA which resulted in telomere elongation in CHLA-90 cells and SK-N-SH cells rendered these cells more resistant against radiation. Knock-down of TA by a dominant negative mutant in TA-positive SK-N-SH cells induced a more radiosensitive phenotype. These observations are in good accordance with studies, which observed an enhanced radiosensitivity of mice whose telomeres were shortened due to a mutant hTERT [8,12,30,31].

Continued inhibition of TA gradually erodes telomeres and leads to chromosome instabilities. Irradiation induces DNA damage and it is likely that eroded and instable chromosomes are targeted more easily by irradiation.

Though the anticancer drugs tested also induce DNA damage, this concept obviously does not apply that strictly to the combination of chemotherapy and telomerase inhibition. TA knock down increased the sensitivity to ifosfamide of SK-N-SH cells, but decreased the sensitivity to cisplatin, doxorubicin, and etoposide. These effects of TA-inhibition on chemosensitivity were most prominent after an exposure for 24 h and evened after 96 h. Knock down of TA only reduced the growth of SK-N-SH cells after more than 28 passages. The effects of chemotherapy were studied when the telomeres already shortened but before they reached their critical length. At this time point the proliferation rate between not-transfected, S11hTWT-, S11IN- and S11hTDNIN-transfected cells did not differ. Thus, the observed effects of TA-inhibition on chemosensitivity were not influenced by different proliferation rates. A number of studies addressed the effect of TA inhibition on radio- and chemosensitivity. While radiosensitisation by telomerase inhibition has been unambiguously reported in literature, the effects of chemotherapy combined with telomerase inhibition obviously depend on the anticancer drugs and the cell lines used. Chen et al. treated prostate cancer cell lines antisense oligonucleotides and studied the effect of the standard antiproliferative agents, paclitaxel, doxorubicin, etoposide, cisplatin, or carboplatin at the beginning of antisense treatment and after erosion of telomeres. They found no effects of TA inhibition on chemosensitivity at the beginning of antisense treatment. When telomeres were shortened the cells were more sensitive to cisplatin and carboplatin but not to paclitaxel, doxorubicin, and etoposide [32].

However, long telomeres and high telomerase activity are distinct features of highly proliferating cells (e.g. germ cells, stem cells) and are reported to be essential vitality factors of cancer stem cells [33-35]. These cells are defined as a small subpopulation of cancer cells, which have the ability of self-renewing and to produce heterogeneous lineages of cancer cells that comprise the tumor [18]. Should it be proved to be true that these cells are more resistant towards therapeutic regimens, it follows that they can limit the therapeutic outcome and impair long term curability. However, the stem cell marker telomerase influences radiation response and chemoresistance and therefore, could be one potential factor influencing cancer stem cell survival under therapy. The here described construct with telomerase knock-down in combination with other stem cell markers like CD133, CD44/CD24, ALDH-1 and SP may be useable to verify this in further experiments.

## Conclusions

In summary, our results support the concept of telomerase inhibition as an antiproliferative treatment approach for neuroblastomas. Regarding irradiation our data further suggest that telomerase inhibition improves radiation response of neuroblastomas. With respect to the varying effects reported for telomerase inhibition combined with chemotherapy our data complete this picture of drug- and cell line-dependent additive/synergistic or antagonistic effects of telomerase inhibition combined with chemotherapy and suggests positive effects of combinations with certain anticancer drugs. Further experiments should clarify the role of telomerase acticity on the long term curability of radio- and chemotherapy by targeting cancer stem cells which are known to have long telomeres and high telomerase activity.

## Conflicts of interests

The authors declare that they participated in the here listed contributions made to the study and that they have seen and approved the final version. They declare no conflict of interest or financial relationship influencing the conclusions of the work.

## Authors' contributions

SW and CLK have contributed to the same extent to the manuscript and carried out most of the experiments shown here. IDS and TB did parts of the statistical analysis and helped in discussion of data. KLSCH and YB carried out generation of the transformed cell lines. NW participated substancially in the design of this study and BG worked out the study design and carried out the telomer-length experiments. All authors read and approved the final manuscript.
